# Epigenetic chromatin modifiers in barley: IV. The study of barley Polycomb group (PcG) genes during seed development and in response to external ABA

**DOI:** 10.1186/1471-2229-10-73

**Published:** 2010-04-21

**Authors:** Aliki Kapazoglou, Alessandro Tondelli, Dimitra Papaefthimiou, Helen Ampatzidou, Enrico Francia, Michele A Stanca, Konstantinos Bladenopoulos, Athanasios S Tsaftaris

**Affiliations:** 1 Institute of Agrobiotechnology, CERTH, Thermi-Thessaloniki, GR-57001, Greece; 2 CRA-Genomic Research Centre, Fiorenzuola d'Arda, I-29017, Italy; 3 Department of Agricultural and Food Science, University of Modena and Reggio Emilia, I-42100, Reggio Emilia, Italy; 4 Cereal Institute, NAGREF, Thermi-Thessaloniki, GR-57001, Greece; 5 Department of Genetics and Plant Breeding, Aristotle University of Thessaloniki, GR-54124, Thessaloniki, Greece

## Abstract

**Background:**

Epigenetic phenomena have been associated with the regulation of active and silent chromatin states achieved by modifications of chromatin structure through DNA methylation, and histone post-translational modifications. The latter is accomplished, in part, through the action of PcG (Polycomb group) protein complexes which methylate nucleosomal histone tails at specific sites, ultimately leading to chromatin compaction and gene silencing. Different PcG complex variants operating during different developmental stages have been described in plants. In particular, the so-called FIE/MEA/FIS2 complex governs the expression of genes important in embryo and endosperm development in *Arabidopsis*. In our effort to understand the epigenetic mechanisms regulating seed development in barley (*Hordeum vulgare*), an agronomically important monocot plant cultivated for its endosperm, we set out to characterize the genes encoding barley PcG proteins.

**Results:**

Four barley *PcG *gene homologues, named *HvFIE*, *HvE(Z), HvSu(z)12a*, and *HvSu(z)12b *were identified and structurally and phylogenetically characterized. The corresponding genes *HvFIE*, *HvE(Z), HvSu(z)12a*, and *HvSu(z)12b *were mapped onto barley chromosomes 7H, 4H, 2H and 5H, respectively. Expression analysis of the *PcG *genes revealed significant differences in gene expression among tissues and seed developmental stages and between barley cultivars with varying seed size. Furthermore, *HvFIE *and *HvE(Z) *gene expression was responsive to the abiotic stress-related hormone abscisic acid (ABA) known to be involved in seed maturation, dormancy and germination.

**Conclusion:**

This study reports the first characterization of the *PcG *homologues, *HvFIE, HvE(Z)*, *HvSu(z)12a *and *HvSu(z)12b *in barley. All genes co-localized with known chromosomal regions responsible for malting quality related traits, suggesting that they might be used for developing molecular markers to be applied in marker assisted selection. The *PcG *differential expression pattern in different tissues and seed developmental stages as well as in two barley cultivars with different seed size is suggestive of a role for these genes in barley seed development. *HvFIE *and *HvE(Z) *were also found to be induced by the plant hormone ABA implying an association with ABA-mediated processes during seed development, germination and stress response.

## Background

Epigenetic regulation of gene expression plays a central role in eukaryotic development and takes place through modulation of chromatin structure. Highly organized chromatin consisting of DNA wrapped around histone proteins in nucleosomal structures can switch between relaxed and condensed states associated with transcriptional activity and transcriptional repression, respectively [1]. This is accomplished though cytosine methylation of DNA and post-translational modifications of nucleosomal histone tails. Histone modifications include changes in methylation, acetylation, and phosphorylation on specific lysines and arginines residues. Generally methylation of histone 3 on lysine 9 and 27 (H3K9, H3K27) leads to a repressive chromatin state correlating with gene silencing whereas methylation of histone 3 on lysine 4 (H3K4) leads to a permissive chromatin state correlating with gene activation [2]. Histone acetylation and deacetylation result in active and silent chromatin forms, respectively [3]. This kind of epigenetic regulation operates on various aspects of plant growth and development, including seed formation and stress responses (reviewed in [4,5]).

In flowering plants, the seed is formed through the process of double fertilization. Fertilization of the egg cell by a sperm cell from the male gametophyte generates the diploid embryo from which the organs, tissues and shoot meristems of the plant will be generated. Fertilization of the adjacent central cell by a second sperm cell forms a triploid endosperm, in angiosperms, which supports embryo growth and development by producing storage proteins, lipids, and starch [6].

Bypass of double fertilization is observed in natural apomicts or after mutagenesis of specific gene loci. In *Arabidopsis *mutations of the *FIS *(Fertilization Independent Seed) class of genes: *FERTILIZATION INDEPENDENT SEED (FIE), MEDEA (MEA), FERTILIZATION INDEPENDENT SEED2 (FIS2), MULTICOPY SUPRESSOR OF IRA1 (MSI1) and BORGIA (BGA)*, result in the formation of seed-like structures in the absence of double fertilization that eventually collapse [7-9]. The phenotype of *fie, mea, fis2 *and *msi1 *mutants is accompanied by another impressive characteristic, that is, endosperm overproliferation [9,10]. Endosperm overproduction and seed size increases are also observed in crosses where extra paternal genomes or hypomethylated maternal genomes are used [11]. Another feature of the *fis *phenotype is that when the female gametophyte carrying a mutation in a *fis *locus is fertilized by a wild type plant, the seed finally collapses indicative of a lethal maternal effect of the *fis *mutation and a gametophytic maternal control over seed development by *FIS *genes [7,8]. This is partially attributed to parental imprinting of *FIE, MEA *and *FIS2 *where only the maternal allele is expressed during the early stages of seed development [12-14].

The common phenotypic effects of the *fis *mutants are explained by the shared function of the FIS proteins in a PcG complex. The participation of these proteins in a PcG complex was first proposed by [10] and it is now well established that FIE, MEA, FIS2 and MSI1 form the so called FIE/MEA complex, one of the three PRC2 (Polycomb Repression Complex 2) homologues in *Arabidopsis*, regulating seed formation [15].

The PRC2 complex also known as EXTRA SEX COMBS-ENHANCER OF ZESTE (ESC-E(Z)) complex is one of three PcG complexes in *Drosophila*. PRC2 has been shown to play a role in the epigenetic silencing of target genes involved in cell growth and proliferation and in early development both in *Drosophila *and mammals. Its function involves H3K27 methyltransferase activity [16]. Methylation of lysines 9 and 27 of histone 3 serve as epigenetic marks of transcriptional silent loci. The PcG/PRC2 complex in *Drosophila *contains four essential subunits: two WD40 proteins, named EXTRA SEX COMBS (ESC), and P55 containing the WD40 domain involved in protein-protein interactions, a zinc finger protein, SUPRESSOR OF ZESTE 12 [Su(z)12], and a SET-domain protein responsible for methyltransferase activity, ENHANCER OF ZESTE E(Z) [E(Z)] [17,18].

Different variants of the PRC2 complex have been identified in plants depending on developmental stage, the best studied one being the FIE/MEA complex which regulates the initiation of seed development [18]. It consists of FIE, MEA, FIS2 and MSI1, which are homologous to the *Drosophila *ESC, E(Z), Su(z)12, and P55, respectively [19,20,13,10]. In *Arabidopsis *all PcG complexes are predicted to contain the FIE and MSI1 subunits. Depending on cell type and function the different PcG complexes contain one of the three homologues of the *Drosophila *EZ protein, MEA, CURLY LEAF (CLF) or SWINGER (SWN), and one of the three homologues of the *Drosophila *Su(z)12 protein, EMBRYONIC FLOWER 2 (EMF2), FERTILIZATION INDEPENDENT SEED 2 (FIS2), and VERNALIZATION2 (VRN2), respectively. The EMF2-CLF/SWN-FIE-MSI1 complex has been suggested to play a role in suppressing the transition from vegetative development to flowering and flower organ formation [15,21] and the VRN2-CLF/SWN-FIE-MSI1 has been implicated in the regulation of the process of vernalization [15,22,23]. EMF2, FIS2 and VRN2, the three Su(z)12-like proteins in *Arabidopsis*, contain a classical C_2_H_2 _zinc finger similar to the fingers found in sequence-specific DNA-binding proteins and another stretch of amino acids, located C-terminal to the zinc finger, that is conserved between DsSu(z)12, HsSu(z)12, EMF2, VRN2 and FIS2 and has been termed **VEFS **box (**V**RN2-**E**MF2-**F**IS2-**S**u(z)12 box) [24].

Homologues of the PcG proteins of the FIE-E(Z) complex have been identified in other plant species including monocots, indicating their involvement in a conserved regulatory mechanism among all higher plants [17].

Three target genes for the *Arabidopsis *FIS2-FIE-MEA-MSI1 complex operating in seed development have been identified so far. The first one is *PHERES1(PHE1)*, a type I MADS box gene [25] which is parentally imprinted in the developing seed, with the maternal allele being silenced and the paternal allele expressed in specialized endosperm cells during the first two days after fertilization [26,27]. The second target is the *MEA *gene itself, demonstrating an autoregulating mechanism for the imprinting of a PcG epigenetic silencer [28,29]. The third one is *FUS3*, a transcription factor belonging to the plant-exclusive B3 domain TF family, specifically expressed in seeds during the seed filling phase [30].

Seed development, dormancy and germination are under the control of plant hormones, in particular ABA and gibberellic acid (GA). ABA inhibits germination and is required for the acquisition of dessication tolerance and entry into seed dormancy acting antagonistically to GA during these processes [31,32]. It is also involved in various aspects of plant growth and development including abiotic stress tolerance. Histone modifications have been suggested to play a major role in ABA-mediated processes such as seed development, germination and abiotic stress adaptation [4]. For example, in leaves of *Phaseolus vulgaris*, externally supplied ABA has been shown to induce methylation of H3K4 and acetylation of H3K14 and H4K5 (indicative of gene activation) of a chromatin region around the *phaseolin *promoter, and ectopic expression of the otherwise silent in leaves *phaseolin *gene (encoding a major seed storage protein) [33]. Likewise, ABA-treated seeds of an *Arabidopsis *mutantof PICKLE (a chromatin remodelling factor mediating suppression of embryonic identity upon germination) showed high expression levels of the ABA-induced transcription factors, ABI3 and ABI5, involved in seed filling. This correlated with reduced H3K9 and H3K27 methylation at ABI3 and ABI5 promoters, in the ABA treated mutant seeds than in wild type seeds [34]. In another study, ABA treatment caused severe reduction in expression of the *Arabidopsis *histone deacetylase gene, *AtHD2C*, whereas overexpression of *AtHD2C *resulted in enhanced abiotic stress tolerance and both repression or induction of several ABA-responsive genes [35]. Recently, ABA was suggested to be a positive regulator of SWI3B, a subunit of the chromatin remodelling complex SWI/SNF, as in *Arabidopsis swi3b *mutant seedlings exposed to external ABA there was reduced expression of ABA-responsive genes [36]. Furthermore, ABA or an ABA signal was suggested to affect epigenetic states, the DNA replication machinery and chromatin mediated gene expression in *Arabidopsis *seedlings [37].

Despite the extensive studies on epigenetic regulation of seed development in *Arabidopsis*, knowledge on epigenetic regulation through DNA and chromatin modulation in cereal seed development is only now starting to emerge. The expression of a large number of genes encoding structural proteins, metabolic enzymes, transcription factors and DNA and chromatin modulators during seed development and germination has been investigated in barley, through large-scale transcriptome analysis [38]. Very recently, as part of our efforts to study epigenetic control mechanisms during seed development in barley, we isolated members of the two families of histone deacetylases, the plant specific HD2 family and the RPD3/HDA1 family as well as members of the histone acetyltransferase families, GCN5, MYST, ELP3, respectively, and investigated their expression at different seed developmental stages, in different grain-size cultivars and in response to external hormonal stimuli [39-41].

Considering the economic importance of cereal crops such as barley, rice, wheat and maize, we report here the characterization of the barley *PcG *gene homologues. More specifically, a *FIE *homologue, termed *HvFIE*, an *E(Z) *homologue, termed *HvE(Z)*, and two *Su(z)12-like *homologues, termed *HvSu(z)12a *and *HvSu(z)12b*, respectively, were characterized. The mapping position of *HvFIE, HvE(Z)*, *HvSu(z)12a*, and *HvSu(z)12b *on different barley chromosomes was assigned, and their expression was examined in different tissues and seed developmental stages and in barley cultivars which differ in seed size and weight. Furthermore, the expression of *HvFIE, HvE(Z) *and *HvSu(z)12b *was investigated after treatment with the plant hormone ABA, a modulator of gene expression during developmental and environmental changes, operating often through epigenetic modifications of target genes.

## Methods

### Plant material

Commercial barley cultivars, Caresse, Carina and Ippolytos, differing in seed size and weight were planted in the field and were the source of RNA for expression analysis. For Caresse, the weight of 1000 grains is 50-55 gr, and 98% of seeds have diameter longer than 2.5 mm, for Carina the weight of 1000 grains is 36-40 gr and 60-65% of seeds have diameter longer than 2.5 mm whereas for Ippolytos, seeds weigh 25-31 gr per 1000 grains and only 35-45% of seeds have diameter longer than 2.5 mm http://www.cerealinstitute.gr. The Oregon Wolfe Barley Dominant (OWB-D), Oregon Wolfe Barley Recessive (OWB-R), L94 and Vada parental barley lines were used for polymorphism detection and mapping.

### Hormonal treatment

Seven-day-old seedlings (Caresse) grown in a growth chamber (16 hours (h) light, 8 h darkness, at 22°C) were sprayed with 100 μM ABA, (abscisic acid +/- *cis*, *trans*-ABA, SIGMA). Aerial parts of plants were collected at 6 h and 24 h after treatment and immediately stored in liquid nitrogen. Aerial parts from five plants were pooled together for RNA extraction for each time point. Control plants were sprayed with water plus 0.2% Tween.

### Identification of barley PcG gene homologues and protein analysis

The barley *FIE *and *E(Z) *homologues were identified by searching the barley EST database at the TIGR Web site http://www.tigr.org. Blast searches were performed using the complete cDNA sequence of *Arabidopsis FIE, MEA, FIS2*. Two tentative consensus sequences of barley ESTs in the TIGR web site, TC134355 and TC140937, were identified, coding for genes which are homologous to the *Arabidopsis FIE *and *MEA*, respectively. A blastn search on the NCBI Web site http://blast.ncbi.nlm.nih.gov/Blast.cgi using TC140937 identified a full length E(Z) mRNA sequence (AK251149) from the BARLEY DB http://www.shigen.nig.ac.jp/barley/. Three complete mRNA sequences encoding *Su(z)12-like *homologues were identified in GenBank with accession numbers AB085817, AB085818, AB085819, respectively. Homology searches were performed with a BLAST algorithm [42] against the DDBJ/EMBL/GenBank database. Sequence alignments were generated with the Clustal W Method [43] of MegAlign (LaserGene software) and processed with the Bioedit software. The sequences and accession numbers used for protein sequence analysis are shown in Table 1. The phylogenetic tree was calculated using MEGA 3.1 software [44] by the Neighbor-Joining Method with p-distance correction [45]. For E(Z), the Bayesian inference of phylogeny was applied with the use of MrBayes 3.1.2 [46]. Four Metropolis-coupled Markov chain Monte Carlo chains were run on one million generations, with sampling every 100 generations. After discarding the first 5,000 unstable trees as burn-in, a majority-rule consensus tree was visualized and edited with TreeView 1.6.6 [47].

**Table 1:** Protein sequences used for alignments and phylogenetic tree construction.

Organism name	Gene name	Accession number
*Arabidopsis thaliana*	AtFIE	AF129516

*Catalpa speciosa*	EgrFIE	AY150282

*Eucalyptus grandis*	CspFIE	AY150283

*Hordeum vulgare*	HvFIE	TC134355

*Oryza sativa nipponbare*	OsnFIE2	BAD03073

*Oryza sativa japonicum*	OsjFIE	BAD03075

*Sorghum bicolor*	SbiFIE1	AAS82595

*Sorghum bicolor*	SbiFIE2	AAS82596

*Zea mays*	ZmFIE1	AY061964

*Zea mays*	ZmFIE2	AY061965

		

*Arabidopsis thaliana*	AtSWN	NP_567221

*Arabidopsis thaliana*	AtCLF1	AAC23781

*Arabidopsis thaliana*	AtMEA	AAC39446

*Hordeum vulgare*	HvEZ	AK251149

*Oryza sativa indica*	OsiEZ1	BAD69169

*Oryza sativa japonicum*	OsjEZ	CAD18871

*Petunia hybrida*	PhCLF1	BAC84950

*Petunia hybrida*	PhCLF2	BAC84951

*Petunia hybrida*	PhCLF3	BAC84952

*Triticum monococcum*	TmEZ	ABI94364

*Zea mays*	MEZ1	AF443596

*Zea mays*	MEZ2	AF443597

*Zea mays*	MEZ3	AF443598

		

*Arabidopsis thaliana*	AtEMF2	BAB58956

*Arabidopsis thaliana*	AtIEMF2-isoform	Q8L6Y4-2

*Arabidopsis thaliana*	AtFIS2,	ABB84250

*Arabidopsis thaliana*	AtFIS2b	AAD09104

*Arabidopsis thaliana*	AtVRN2	AAL32135

*Dendrocalamus latiflorus*	DendrEMF2	ABB77210

*Hordeum vulgare*	HvSu(z)12a (HvEMF2a)	BAD99130

*Hordeum vulgare*	HvSu(z)12b (HvEMF2b	BAD99131

*Hordeum vulgare*	HvSu(z)12c (HvEMF2c)	BAD99132

*Oryza sativa*	OsEMF	AAQ84239

*Oryza sativa japonica*	OsVEF	AP005912

*Oryza sativa*	OsEMF-like	Q7XSN0

*Triticum aestivum*	TmEMF	AY887064

*Yucca filamentosa*	YfEMF2	ABD85300

*Zea mays*	ZmEMF2-like (VEF101)	AAO84022

*Zea mays*	ZmEMF-like	ABC69154

*Drosophila melanogaster*	DmSu_z_12	AAF73149

*Mus musculus*	MmSu(z)12	BAC65495

*Homo sapiens*	HsSu(z)12	BAA09931

### Mapping of barley PcG genes

Genomic DNAs extracted from 94 'OWB-Dominant' x 'OWB-Recessive' [48] doubled haploid lines and from 103 'L94' x 'Vada' [49] recombinant inbred lines were used for linkage analyses. Specific PCR primer pairs were designed based on the nucleotide sequences of the *PcG *genes by using the software Primer3 [50] (Table 2). PCR amplifications were performed in a 20 μl final volume containing: 40 ng of genomic DNA as template, 1× Promega PCR buffer, 1.5 mM of MgCl_2_, 5% DMSO, 0.25 mM of each dNTP, 0.4 μM of each primer, and 1 U of Taq DNA Polymerase (Promega, Madison USA). Reactions were incubated for 2 min at 94°C, followed by 40 cycles of 94°C for 30 s, 55°C for 30 s, 72°C for 1 min, and a final extension of 72°C for 7 min. PCR products were purified using the Wizard SV Gel and PCR Clean-Up System (Promega, Madison USA), and sequenced on both strands to confirm their identities, then sequences were aligned using the Staden package http://staden.sourceforge.net to identify Single Nucleotide Polymorphisms (SNPs). Inspection of the restriction enzyme cleavage sites with the software WebCutter http://www.firstmarket.com/cutter) revealed *Hind*III and *DdeI *restriction enzyme cleavage polymorphisms in the sequenced *HvE(Z) *and *HvSu(z)12a *PCR products, respectively. For *HvSu(z)12b *and *HvFIE *sequence polymorphisms identified between the mapping parents were not within restriction sites, so PCR markers were made by introducing a restriction site (*Rsa*I and *Dde*I, respectively) into the PCR product using the dCAPS strategy [51]. According to manufacturer's instructions, 5 μl of PCR product were incubated for 1.5 h with 1 U of the corresponding restriction enzyme, 1× reaction buffer and 0.1 mg/ml of bovine serum albumin, and then separated on a standard 2% agarose gel. The positions of the genes in the barley genome have been determined with high resolution by integrating the new PcG data with data that are publicly available. Linkage analysis was performed with the software JoinMap 4.0 [52] by adding the *HvFIE *and *HvEZ *markers to the 'OWB-D' x 'OWB-R' map [53], http://wheat.pw.usda.gov/ggpages/maps/OWB), and the *HvSu(z)12a *and *HvSu(z)12b *markers to the 'L94' x 'Vada' map [49], http://wheat.pw.usda.gov/report?class=mapdata&name=Barley,+L94+x+Vada,+2006).

**Table 2:** Primers used for expression analysis and mapping.

Gene	Primer	Sequence (5'-3')	Tm (°C)	Fragment (bp)
	Expression			
*HvFIE*	HvFIE F1	GTGCCCGATTGTGACATTTGGTTT	70	264
	HvFIE R1	CACGGCGGTTTTTGCTTCCTTC	68	
*HvE(Z)*	HvE(Z) F1	AAGCATCTGGTTCTCATCGTC	62	293
	HvE(Z) R1	TGGTCCACCTCAACAAGTAGA	62	
*HvSu(z)12a*	HvSu(z)12a F1	TCGTGACTCGGTAGGAAACATTG	68	261
	HvSu(z)12a R1	CCAATGTTAATAGGTGTTTCAAG	62	
*HvSu(z)12b*	HvSu(z)12b F1	GTATATGAGTTGAGCATAGTGC	62	278
	HvSu(z)12b R1	TTCAGATGGATTTCATTACAGC	60	
*HvSu(z)12c*	HvSu(z)12c F	ATGTGCCGTCAACCGTCCACG	68	463
	HvSu(z)12c R	CTCCAGCTTGCCCTTGTGAAG	66	
*HvActin*	Barley Actin F	ATGGCTGACGGTGAGGACATCCAGCC	72	1111
	Barley Actin R	TCAGAAGCACTTCCTGTGGACGATCGC	71	
	HvActin F	GCCGTGCTTTCCCTCTATG	60	235
	HvActin R	GCTTCTCCTTGATGTCCCTTA	62	
*HVA22*	*HVA22 F*	TGGCGCTCCCGCAGTTCAAG	64	123
	*HVA22R*	CCTTGAGTATGTGCACCTTGTG	62	
				
	Mapping			
*HvFIE*	HvFIE F2	GTGGGAGCCAAAAACAAAAG	60	262
	HvFIE R2	GGCGTCACAGGCTAAGTAAGG	61	
*HvE(Z)*	HvE(Z) F2	AAATCAGATGTTGCAGGATGG	60	550
	HvE(Z) R2	GCTCCCTTGCATAGATACCAAC	61	
*HvSu(z)12a*	HvSu(z)12a F2	ACTCGTGCAGAACCCAAGAC	60	576
	HvSu(z)12a R2	ACCCATCCCAGCTACAACAG	60	
*HvSu(z)12b*	HvSu(z)12b F2	ATATTCCTTGGGCCTGTGAG	59	308
	HvSu(z)12b R2	TGGAACAGAACACGACGAAG	54	

### RNA isolation and first strand cDNA synthesis

Total RNA was isolated from roots, shoots, apical meristems, first leaves of seedlings, pistils, stamens, whole flowers before fertilization (immature flower, IMF), seeds 1-3, 3-5, 5-10, 10-15 days after flowering (DAF), and aerial parts after hormonal treatment, respectively, using TRI REAGENT (SIGMA) according to the instructions of the manufacturer. First strand cDNA synthesis was performed using 1.0 μg total RNA, 0.5 μg 3' RACE Adapter primer 5'-GGCCACGCGTCGACTAGTAC (T)_17_-3' (Invitrogen), 1 mM dNTPs and 200 U M-MuLV reverse transcriptase (Invitrogen,) in 20 μL total volume, according to the specifications of the manufacturer.

### Expression analysis of barley PcG genes

Qualitative RT-PCR was used for examining the expression of the *HvFIE, HvE(Z)*, *HvSu(z)12a, HvSu(z)12b and HvSu(z)12c *barley homologues in different tissues. cDNAs produced from 1 μg of total RNA from roots, shoot meristems, young shoots, leaves, stamens, and pistils before fertilization from a medium-seed-size cultivar, Carina, were used. The primer pair Barley Actin F/Barley Actin R, amplifying a 1111 bp fragment of the barley actin coding gene [AY145451], was used as internal control for successful cDNA synthesis. The PCR conditions for actin were: initial denaturation at 94°C for 3 min, then 30 cycles at 94°C for 30 s, 70°C for 1 min, 72°C for 2 min and final extension at 72°C for 15 min. The PCR conditions for the *HvFIE *were: initial denaturation at 94°C for 2 min, then 30 cycles of 94°C for 30 sec, 53°C for 30 sec, 72°C for 40 sec and final extension at 72°C for 10 min. The same thermocycler program was used for the fragment amplification of the other four genes, except for the annealing step which was 57°C for *HvE(Z)*, *HvSu(z)12b*, and *HvSu(z)12a*, and 62°C for *HvSu(Z)12c *respectively.

Quantitative real-time RT-PCR was performed with cDNA synthesized from 1 μg of total RNA from immature flowers, seeds 1-3 DAF, 3-5 DAF, 5-10 DAF, and 10-15 DAF from Caresse and Ippolytos, respectively, and aerial parts of Caresse seedlings after ABA treatment. For each sample a reaction was set up in a PCR reaction mix (20 μl) containing 5 μl of the 1:200 diluted cDNA, 0.25 μM of each primer and 1× Platinum SYBR Green qPCR Supermix-UDG (Invitrogen, Paisley, UK) and using an Opticon 2 (MJ Research, Waltham, MA) real-time PCR system and the Opticon Monitor 3 software package (MJ Research). Each reaction was performed in triplicates. General thermocycler conditions were 50°C for 2 min, 95°C for 2 min, then 42 cycles of 95°C for 30 sec, annealing [*HvE(Z)*: 56°C; *HvFIE*: 57°C; *HvSu(z)12b*: 54°C, respectively] for 30 sec, 72°C for 30 sec, then 72°C for 5 min and plate read at 82°C. To identify the PCR products a melting curve was performed from 65°C to 95°C with observations every 0.2°C and a 10-s hold between observations. Relative quantification and statistical analysis were performed with the REST software [54] and using actin as the reference gene with HvActinF/HvActinR as primers. The barley gene *HVA22*, which is known to be induced by ABA [55] was used as a positive control. All primers used in expression analysis correspond to non-conserved regions and are shown in Table 2.

## Results

### Identification of barley PcG gene homologues and analysis of putative proteins

**Figure 1:**
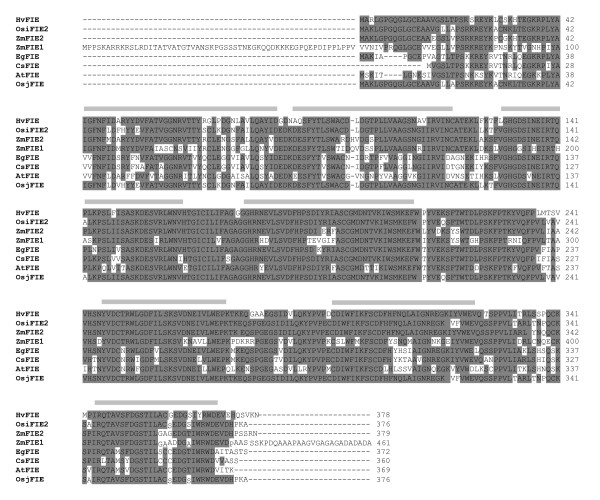
**Alignment of the amino acid sequences of the FIE homologues in different plants**. The sequences and accession numbers used for protein sequence analysis are shown in Table 1. Identical and similar amino acid residues are shaded in dark grey and light grey, respectively. WD 40 repeats are marked above the sequences with grey lines.

A blast search of the *Hordeum vulgare *EST database in TIGR http://www.tigr.org detected an EST (TC134355) corresponding to *FIE *and an EST (TC140937) corresponding to *E(Z)*, respectively. TC134355 contains a full length cDNA sequence (1623 nt translating to 378 aa) of a barley *FIE *homologue, which we termed *HvFIE*. Amino acid sequence alignment of plant FIE homologues revealed that the HvFIE putative protein belongs to the highly conserved FIE family and that it contains the seven characteristic WD40 repeats (Figure 1).

**Figure 2:**
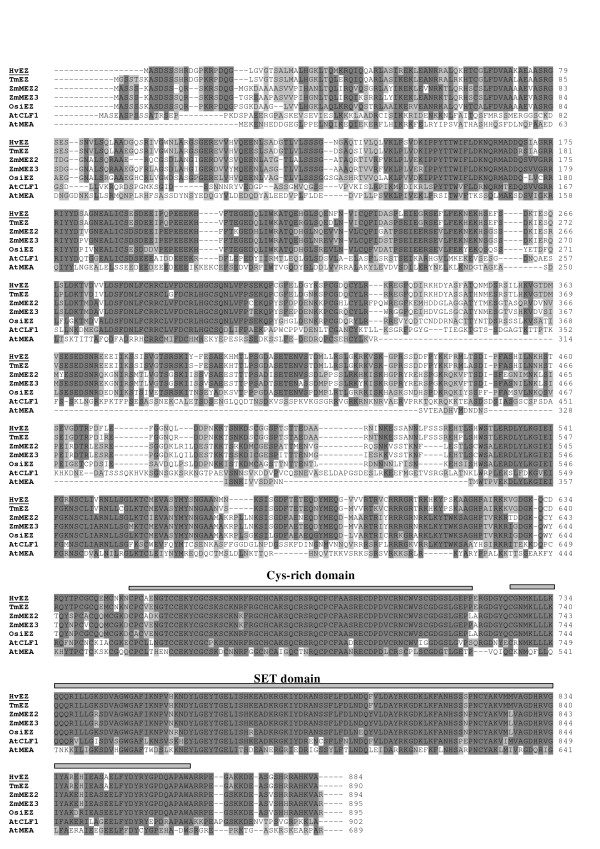
**Alignment of the amino acid sequences of the E(Z) homologues from different plants**. Sequences and accession numbers are shown in Table 1. Identical and similar amino acid residues are shaded in dark grey and light grey, respectively. The cysteine rich domain and the SET-domain are shown above the sequences with hatched and gray lines, respectively.

TC140937 contains a partial cDNA sequence (1151 nt) corresponding to a barley *E(Z) *homologue which we termed *HvE(Z)*. A tblastn search with this sequence revealed a full length mRNA sequence (AK251149) from the BARLEY DB http://www.shigen.nig.ac.jp/barley/ which contains 3092 nt encoding 884 aa of an E(Z) homologue. The putative E(Z) protein from barley, HvEZ, contains the characteristic SET-domain and the cysteine-rich region as shown in the alignment of the E(Z) amino acid sequences from different organisms (Figure 2). The barley protein has 94% identity with the E(Z) protein from wheat (TmEZ), and 70% identity with the EZ2 and EZ3 proteins from maize (MEZ2, MEZ3).

**Figure 3:**
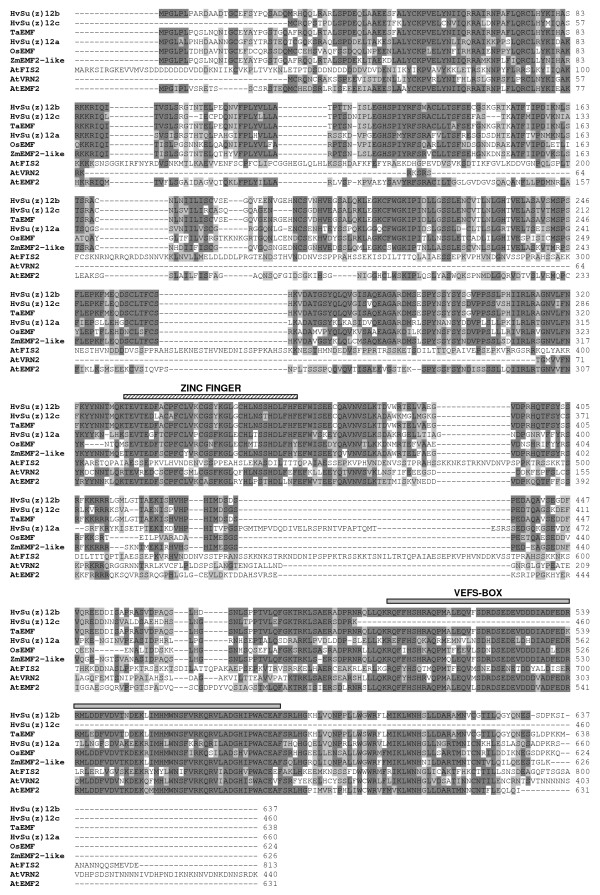
**Alignment of the amino acid sequences of Su(z)12 protein homologues from different plants**. Sequences and accession numbers are shown in Table 1. Identical and similar amino acid residues are shaded in dark grey and light grey, respectively. The Zinc-finger domain and the VEFS box are indicated by hatched and gray lines, respectively.

A search in GenBank identified three complete mRNA sequences from barley coding for Su(z)12-like homologues with accession numbers AB085817, AB085818, and AB085819 which we named *HvSu(z)12a, HvSu(z)12b*, and *HvSu(z)12c*, respectively. These sequences encode putative proteins of 660 aa, 637 aa, and 460 aa, respectively. They all contain the characteristic zinc finger domain found in VEFS proteins, and the HvSu(z)12a, HvSu(z)12b putative proteins also contain the conserved VEFS domain as shown in the VEFS protein alignment (Figure 3). The HvSu(z)12c amino acid sequence terminates before the VEFS domain but this could be due, in part, to a sequencing problem at the 3' end of the particular cDNA as the nucleotide sequence of AB085819 contains unexplained repeats. Because of this problem we decided not to study HvSu(z)12c any further. HvSu(z)12a and HvSu(z)12b share 58% identity with each other. HvSu(z)12b is highly similar to a wheat EMF protein TaEMF, with 93% identity.

**Figure 4:**
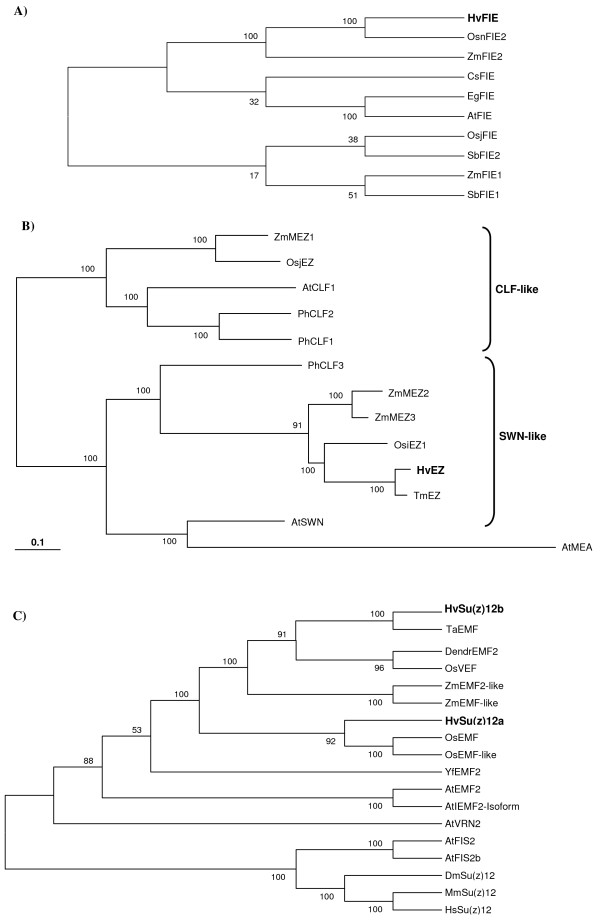
**Phylogenetic analysis of PcG proteins**. Phylogenetic trees of PcG homologues from different organisms. A) FIE. B) E(Z). C) Su(z)12. Barley sequences are in bold. Sequences and accession numbers are shown in Table 1.

A phylogenetic analysis of FIE homologues in plants showed that HvFIE is more closely related to other cereal FIE proteins, specifically, ZmFIE2 from maize and OsnFIE2 from rice (Figure 4A). Similarly, phylogenetic analysis of E(Z) proteins from monocots and dicots showed that HvEZ groups out together with TmEZ, MEZ2, MEZ3 and OsiEZ1, whereas AtCLF, MEZ1, OsjEZ form another cluster and AtSWN and AtMEA are more distant to both groups (Figure 4B). A phylogenetic analysis of various VEFS proteins from different organisms (Figure 4C) showed that HvSu(z)12b is more closely related to EMF proteins from wheat, dendrocalamus, oryza and maize, whereas HvSu(z)12a forms a cluster with two other EMF proteins from rice. They are both more distantly related to the Arabidsopsis EMF, FIS2, and VRN proteins and their isoforms, and from the animal Su(z)12 homologues (*Drosophila*, mouse, and human) which cluster together.

### Mapping of barley PcG genes

**Figure 5:**
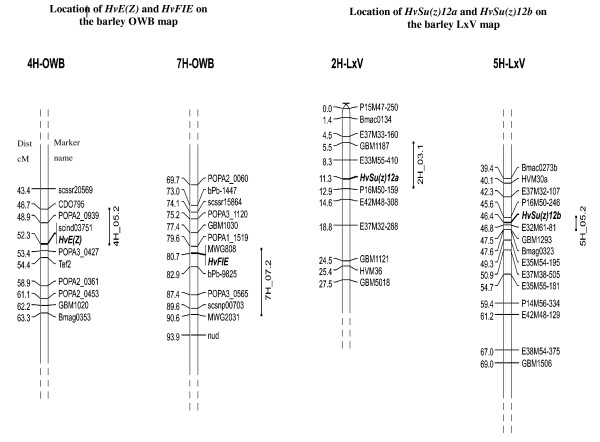
**Mapping of barley *PcG *genes**. Chromosomal position of barley *HvE(z) *and *HvFIE *genes on the OWB molecular linkage map and *HvSu(z)12a *and *HvSu(z12)b *on the LxV molecular linkage map. Only non co-segregating markers are reported, with distances in Kosambi cM. The BIN where each PcG gene has been mapped is indicated following the notation of [49] that integrates data from 6 segregating populations including both the OWB and the LxV maps.

*Hind*III cleaved amplified polymorphic sequence (CAPS) and *Dde*I derived cleaved amplified polymorphic sequence (dCAPS) markers were developed for the mapping of *HvE(Z) *and *HvFIE *on the OWB molecular linkage map, respectively. Linkage analysis revealed that *HvE(Z) *maps in a cluster of 43 molecular markers in the centromeric region of chromosome 4H, and *HvFIE *co-segregates with RFLP marker MWG808 and other 52 markers in the pericentric region of chromosome 7H (Figure 5). CAPS (*Dde*I) and dCAPS (*Rsa*I) marker segregation analyses were performed for mapping *HvSu(z)12a *and *HvSu(z)12b *on the LxV linkage map. *HvSu(z)12a *and *HvSu(z)12b *are located on the short arm of chromosome 2H and on the subcentromeric region of the long arm of chromosome 5H, respectively (Figure 5).

### Differential expression of barley PcG genes in different tissues and seed developmental stages and in two cultivars with varying seed size

**Figure 6:**
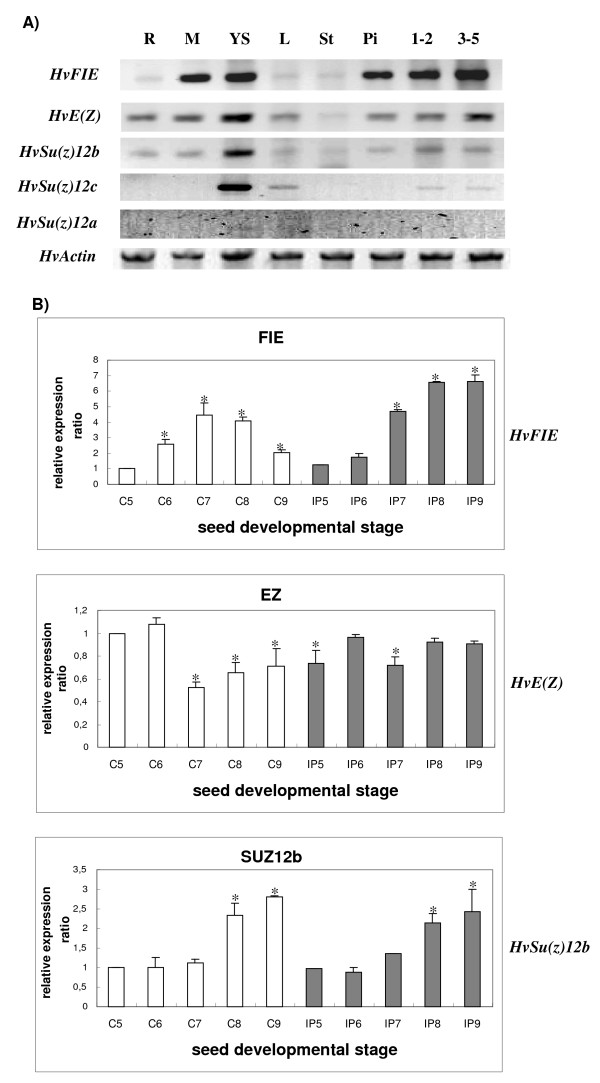
**A) Qualitative RT-PCR expression analysis of barley *PcG *genes**. R, roots; M, meristem; L, Leaves; YS, young shoots; St, stamens; Pi, unfertilized pistils; 1-2, 1-2 DAF; 3-5, 3-5 DAF; *HvActin*, was used as the internal control. Cultivar was Carina (a medium-grain cultivar). DAF, Days after fertilization. **B) Quantitative real-time RT-PCR analysis of *PcG *>genes**. Expression values were normalized to those of *HvActin*. The relative expression ratio of each sample is compared to the control group which was C5 (Caresse immature flowers). C, cultivar Caresse, (white bars); IP, cultivar Ippolytos (grey bars). 5, Immature flower; 6, Seed 1-3 DAF; 7, Seed 3-5 DAF; 8, Seed 5-10 DAF; 9, Seed 10-15 DAF. Data represent mean values from two independent experiments with standard deviations. Values significantly different (P < 0.05) from the control group (C5) are marked with an asterisk.

Qualitative RT-PCR was performed in order to examine the expression of *HvFIE, HvE(Z), HvSu(z)12a *and *HvSu(z)12b *in different tissues, in a medium-seed-size cultivar, Carina (Figure 6A). The *HvFIE *mRNA is abundant in shoot meristems, young shoots, pistils before fertilization and seeds of 1-2 DAF and 3-5 DAF, whereas it is hardly detectable in roots, leaves and stamens. The *HvE(Z) *transcript is abundant in young shoots and 3-5 DAF seeds, moderate in the other tissues, and detectable in stamens. The *HvSu(z)12b *transcript is abundant in young shoots, at moderate levels in 1-2 DAF seeds and at low levels in the other tissues. The *HvSu(z)12c *transcript is abundant in young shoots, present at moderate levels in leaves, at low levels in seeds 1-2 DAF and 3-5 DAF and undetectable in the other tissues examined, whereas the *HvSu(z)12a *transcript is undetectable in all tissues examined except from young shoots where a very faint band is observed.

Quantitative real-time PCR was performed in order to compare the levels of *HvFIE, HvE(Z)*, and *HvSu(z)12b *mRNA accumulation at different stages of seed development and between two barley cultivars with different seed size, Caresse (larger seed size) and Ippolytos (smaller seed size) (Figure 6B). *HvSu(z)12a *was not included in this analysis, owing to its negligible expression. Similarly, *HvSu(z)12c *was excluded due to undetectable or low expression in reproductive tissue and seed, respectively.

*HvFIE *mRNA accumulation exhibits an increase after fertilization, in both Caresse and Ippolytos. In Caresse *HvFIE *transcript levels reach a maximum at 3-5 DAF (C7) with approximately a 5 fold increase as compared to immature flowers (C5) and decrease thereafter to about 50% in 10-15 (C9) DAF. Conversely, in Ippolytos, *HvFIE *exhibits a continuous induction with a pronounced increase up to 7 fold at 10-15 DAF (IP9) as compared to immature flowers (IP5). At 5-10 DAF and at 10-15 DAF, *HvFIE *transcript levels are markedly higher in Ippolytos (approximately 1.5 and 3.5 fold, respectively) as compared to Caresse (Figure 6B).

*HvE(Z) *transcript levels decline after 1-3 DAF (C6) in Caresse, with maximum decrease in 3-5 DAF (2 fold reduction as compared to immature flowers) whereas comparable decrease was not observed in 3-5 DAF (IP7) for Ippolytos. Contrary to Caresse, *HvE(Z) *expression levels in Ippolytos do not show any significant changes in any of the stages before fertilization and throughout seed development.

*HvSu(z)12b *transcript levels are increased after fertilization at the stages 5-10 and 10-15 DAF as compared to immature flowers, both for Caresse and Ippolytos. No significant differences are observed among immature flowers, 1-3 DAF and 3-5 DAF, in either cultivar, and *HvSu(z)12b *levels are comparable between cultivars throughout seed development.

### Expression analysis of barley PcG genes after treatment with ABA

**Figure 7:**
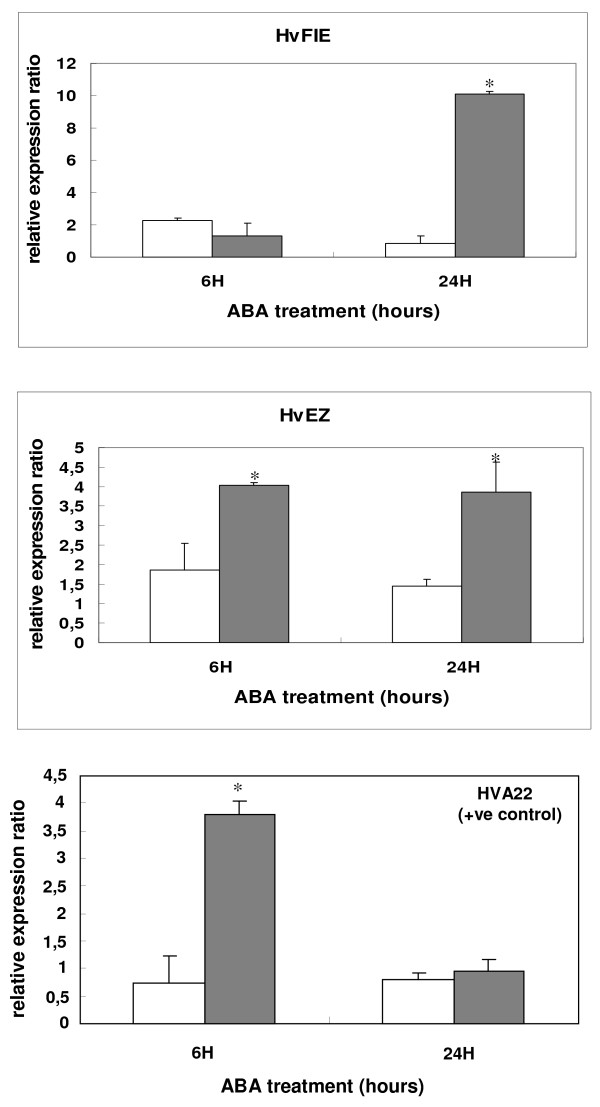
**Expression analysis of barley *PcG *genes after treatment of seedlings with ABA**. Quantitative real-time PCR analysis of *HvFIE *and *HvE(Z) *genes at 6 and 24 h after treatment of Caresse seedlings with 100 μM ABA. Expression values were normalized to those of *HvActin*. Data represent mean values from two independent experiments with standard deviations. Relative expression ratio of each sample was compared to the control group which was untreated plants, 0 h, and was assigned the value of 1. Grey bars, hormone-treated plants for 6 h and 24 h; white bars, no hormone treated plants (mocked with H_2_O/Tween for 6 h and 24 h, respectively). Values significantly different (P < 0.05) from the control group are marked with an asterisk. The barley gene *HVA22 *(known to be induced by ABA) was used as positive control.

Quantitative real time PCR was performed using total RNA from seven-day-old seedlings subjected to exogenous ABA (100 μM) to examine whether *HvFIE *and *HvE(Z*) respond to this seed developmental and stress-related hormone (Figure 7). *HvFIE *showed a striking 10 fold induction at 24 h after ABA spraying of seven-day-old seedlings, as compared to the untreated plants. *HvE(Z*) showed a marked induction of approximately 4 fold, both at 6 h and 24 h after ABA treatment, as compared to the untreated control. Expression of *HvSu(z)12b *did not show any significant changes after ABA treatment (data not shown).

## Discussion

In the current study we present the identification, mapping and expression profiles of the genes encoding PcG chromatin modifiers from barley. This work serves as a first step in understanding the role played by *PcG *genes in the epigenetic mechanisms that control seed formation and stress response in a monocot cereal plant. Unlike dicots, where the endosperm is consumed by the embryo during seed maturation, in monocots such as cereals, the endosperm persists after embryo development is completed and constitutes the major portion of the mature kernel [6,7]. The endosperm of cereal crops like barley, wheat, rice, maize, stores reserves such as proteins, starch, and fatty acids, and represents 60% of the world's food and feed supply. Consequently, understanding and manipulating the molecular mechanisms governing endosperm development for increasing seed yield is of utmost interest for agriculture.

Four barley *PcG *gene homologues encoding the putative PcG proteins HvFIE, HvEZ, HvSu(z)12a and HvSu(z)12b were identified and analyzed. In *Arabidopsis *the respective PcG counterparts are [AtFIE], [AtMEA or AtSWN or AtCLF] and [AtFIS2 or AtEMF2 or AtVRN2]. Three different PcG complex variants can be formed depending on the different combinations of these subunits. The changeable subunits are the MEA and FIS2 homologues whereas FIE is likely to be common in all three complexes. The FIE, MEA, FIS2 proteins form a complex essential for viable seed formation. MEA, CLF, and SWN, constitute the three *Arabidopsis *E(Z) homologues, which cluster into three separate clades, and are members of different PcG complexes with distinct functions. MEA is important for proper seed development and SWN acts redundantly with MEA having a role in preventing endosperm development as well [56]. However according to another report by Spillane et al. 2007 [57], *swn/clf *double mutants in *Arabidopsis *produce normal seeds suggesting that neither SWN nor CLF has a role in seed formation. In addition, SWN and CLF are involved in the vernalization process [23]. It was proposed that these homologues have arisen through an old duplication and separation of *CLF *from the *MEA/SWN *lineage, which occurred before the divergence of monocots and dicots, whereas a more recent duplication produced the separation between *MEA *and *SWN *in only a few species [57]. No direct orthologues of MEA have been found in any other species than *Arabidopsis*. E(Z) homologues identified in maize and rice [58-60], two monocots with sequenced genomes, cluster in two clades: ZmMEZ2, ZmMEZ3 and OsiEZ1 form the so-called SWN-like clade, and ZmMEZ1 and OsjEZ (also named OsCLF) form the CLF-like clade. Phylogenetic analysis showed that the barley homologue HvEZ is a close relative of the wheat, rice and maize E(Z) homologues belonging in the SWN-like clade. Unlike *Arabidopsis*, which has at least three *E(Z) *homologues functioning at different stages of the plant's life cycle, a second HvEZ homologue has not been identified in barley as yet. It might be possible that HvEZ is a common member of the different PcG complex variants that may be operating at different stages of barley development. However, this hypothesis awaits sequencing of the barley genome and further experimentation. In the absence of a MEA counterpart it is possible that the role of MEA is played by one of the putative SWN or CLF homologues both in barley and in the other species lacking MEA.

*HvFIE *maps on chromosome 7Hcen which is colinear with rice chromosome 8, where the rice *OsnFIE2 *homologue (Os08g04270) has been also localized. *HvE(Z) *maps on chromosome 4Hcen which is colinear with rice chromosome 3, where the rice *OsiEZ1 *homologue (Os03g19480) is found [60]. Similarly, *HvSu(z)12a *maps on chromosome 2Hshort which is colinear with rice chromosome 4, where the *OsEMF *(Os04g08034) homologue is localized, and *HvSu(z)12b *maps on chromosome 5Hlong which is colinear with rice chromosome 9 where the rice homologue OsVEF (Os09g13630)is localized [60]. These observations are in agreement with the extensive synteny between barley and rice chromosomes.

*HvFIE *and *Hv(E)Z *are located in the centromeric region of chromosome 7H and 4H repectively, where important clusters of malting quality QTLs have been identified by several authors and reviewed by [61]. Noteworthy, using the Oregon Wolfe Barley map [53] that integrates 2383 loci and a comprehensive summary of 154 QTLs of malting quality http://wheat.pw.usda.gov/ggpages/maps/OWB/, it is possibile to relate the high resolution genomic location of the two PcG genes with historical QTL data. *Hv(E)Z *location is coincident with scind03751 and 1.1 cM proximal to Tef2, the significant marker for the beta-Glucanase activity QTL (QBgsg.StMo-4H) identified in the Steptoe x Morex population. *HvFIE *maps to a cluster of OPA and DArT markers that includes MWG808 and is 10 cM proximal to MWG2031, the significant marker for both Grain protein content (QGpc.HaMo-7H) and Kernel plumpness (QKp.HaMo-7H) in the Harrington x Morex population.

Likewise the map position of the two Su(z)12 homologues (*HvSu(z)12a *on the short arm of chromosome 2H and *HvSu(z)12b *close to the centromere of chromosome 5H), is in proximity to genomic regions historically involved in the genetic control of malting quality traits [61]. However, all these gene-QTL relationships cannot be unequivocally established and thus further research is needed to determine the role of PcG in endosperm-related traits.

Qualitative RT-PCR analysis demonstrated that the barley *HvFIE *shows a tissue expression profile similar to that of *AtFIE*, maize *ZmFIE2 *and rice *OsFIE2 *[13,62,58,63,60] in that it is expressed in both vegetative and reproductive tissues. Similarly, the barley *HvE(Z) *is expressed throughout development like the maize homologues *ZmMEZ1, ZmMEZ2 and ZmMEZ3 *[58] and the rice *E(Z) *homologues, *OsiEZ1 *and *OsCLF *(same as *OsjEZ) *[60], whereas *AtMEA *is not expressed in leaves [19,12,64]. The expression pattern of *HvSu(z)12b *is similar to the rice *OsEMF *homologues, being expressed both in vegetative and reproductive tissues [60]. Conversely, *AtFIS2 *is expressed only in the central cell and endosperm [13]. No expression studies on the maize *Su(z)12 *homologues, *ZmEMF-like *and *ZmEMF2-like*, have been reported so far. The differential expression of *AtMEA *and *AtFIS2 *compared to *E(Z) *and *Su(z)12 *homologues from monocots and other dicots, together with the fact that the protein sequences of AtMEA and AtFIS2, are distantly related to the E(Z) and Su(z)12 homologues from cereals and other dicots supports the view proposed by Luo et al. 2009 [60], that the *MEA *and *FIS2 *genes arose only within the Brassicaceae.

In *Arabidopsis*, mutations in the *PcG *genes resulting in downregulation of *AtMEA, AtFIE *and *AtFIS2*, cause seed-like structure formation in the absence of fertilization and the production of non-viable seeds after fertilization. In both cases endosperm overproliferation has been observed. Endosperm overproliferation could be a desirable feature of viable and qualitatively acceptable seeds in crops where the seed and in particular the endosperm is the agronomically important product. In this study we attempted to analyze quantitatively the expression of the *PcG *gene homologues in different seed developmental stages and in two barley cultivars differing in seed size, in order to unravel any association between the expression of these genes with seed development and the size of seed. Quantitative real time PCR expression analysis revealed a differential expression pattern during seed development and significant differences between two barley cultivars with varying seed size for the *PcG *genes. *HvFIE *exhibits a pronounced increase after fertilization for both cultivars, which declines in Caresse after 3-5 DAF, but increases remarkably in Ippolytos (approximately 7 fold) at 5-10 and 10-15 DAF. Interestingly, *HvFIE *transcript levels are markedly higher in Ippolytos at 5-10 and 10-15 DAF than in Caresse by approximately a factor of 1.5 and 3.5, respectively. *HvE(Z) *expression shows a decline in Caresse seeds 3-15 days after fertilization as compared to flowers prior to fertilization, with a marked 2 fold decrease at 3-5 DAF. Conversely, such a reduction is not observed in Ippolytos, where *HvE(Z) *transcript levels do not show any significant changes among seed stages. In barley, endosperm cellularization begins at approximately 4 DAF and ends at 6-8 DAF, when the seed maturation process begins [6]. It might be possible that the differences in *HvE(Z) and HvFIE *expression between the two cultivars during these critical stages of endosperm development are associated with the processes of cellularization and seed filling and ultimately with the size of seed. For *HvSu(z)12b*, transcript levels show an induction after fertilization at 5-10 and 10-15 DAF, implying a role for *HvSu(z)12b *in the cellularization and seed maturation process for both cultivars. Further experiments will be needed to elucidate the precise roles of these *PcG *genes in barley seed development.

The plant hormone ABA, is involved in various plant developmental processes, including seed development, germination and abiotic stress tolerance. In *Arabidopsis*, ABA plays a central signalling role during seed filling, interacting with master regulator genes such as *ABI3 *and *FUS3*, in a gene activation manner [reviewed in [65]]. Interestingly, *FUS3 *is one of the targets of both the *AtCLF *and *AtMEA *genes (acting in vegetative and reproductive stages, respectively) and it is silenced in leaves and closed flowers [30] where it was also shown to contain the repressive mark H3K27me3. In this work we examined the response of *PcG *genes to ABA after exogenous ABA application on seedlings and observed that both *HvE(Z) *and *HvFIE *genes are induced by ABA. The observed increase in *HvE(Z) *and *HvFIE *expression upon ABA treatment suggests that these genes might play a role as epigenetic regulators of ABA-mediated processes in seedlings. Chromatin modulators, such as histone deacetylase genes, have been shown previously to be affected by exogenous ABA in *Arabidopsis*, rice and barley [35,66,39]. This is the first time that an induction of *PcG *genes upon external ABA exposure has been demonstrated. It is possible that the expression of *PcG *genes is also affected by ABA during seed development, to activate or suppress key regulators, as the cell would require.

## Conclusions

In this work we have presented the identification and characterization of four barley *PcG *genes, *HvFIE, HvE(Z), HvSu(z)12a, and HvSu(z)12b*. All genes were mapped on different barley chromosomes and co-localized with known chromosomal regions responsible for malting quality related traits. Should this be confirmed *PcG *genes might be used as molecular markers for marker-assisted selection in breeding programmes. Differential expression of *HvFIE, HvE(Z), and HvSu(z)12b *in different seed developmental stages and in two barley cultivars differing in seed size, suggested a role for these *PcG *genes in seed development. In addition *HvFIE *and *HvE(Z) *were found to be induced by the hormone ABA involved in seed development and stress response. The present work provides a basis for future studies aiming at unraveling the function of barley *PcG *genes and understanding seed development and stress-response epigenetic regulation in barley and other cereals.

## Authors' contributions

AK conceived and designed the experiments, performed the quantitative real time PCR experiments, and wrote the manuscript. DP performed phylogenetic analysis and part of real time PCR experiments and participated in revising the manuscript. AT and EF performed the mapping experiments and participated in the writing of the manuscript. HA performed tissue sampling and qualitative RT-PCR experiments. KB supervised and attended all barley cultivations. MAS designed the mapping experiments and participated in revising the manuscript. AST conceived and directed the whole study, and participated in the writing and revising of the manuscript. All authors read and approved the final manuscript.
